# Attentional Biases Toward Spiders Do Not Modulate Retrieval

**DOI:** 10.1027/1618-3169/a000584

**Published:** 2023-08-17

**Authors:** Lars-Michael Schöpper, Verena Küpper, Christian Frings

**Affiliations:** ^1^ Department of Cognitive Psychology, University of Trier, Germany

**Keywords:** S-R binding, visual attention, attentional biases, spider fear

## Abstract

**Abstract.** When responding to stimuli, response and stimulus’ features are thought to be integrated into a short episodic memory trace, an event file. Repeating any of its components causes retrieval of the whole event file leading to benefits for full repetitions and changes but interference for partial repetitions. These binding effects are especially pronounced if attention is allocated to certain features. We used attentional biases caused by spider stimuli, aiming to modulate the impact of attention on retrieval. Participants discriminated the orientation of bars repeating or changing their location in prime-probe sequences. Crucially, shortly before probe target onset, an image of a spider and that of a cub appeared at one position each – one of which was spatially congruent with the following probe target. Participants were faster when responding to targets spatially congruent with a preceding spider, suggesting an attentional bias toward aversive information. Yet, neither overall binding effects differed between content of preceding spatially congruent images nor did this effect emerge when taking individual fear of spiders into account. We conclude that attentional biases toward spiders modulate overall behavior, but that this has no impact on retrieval.


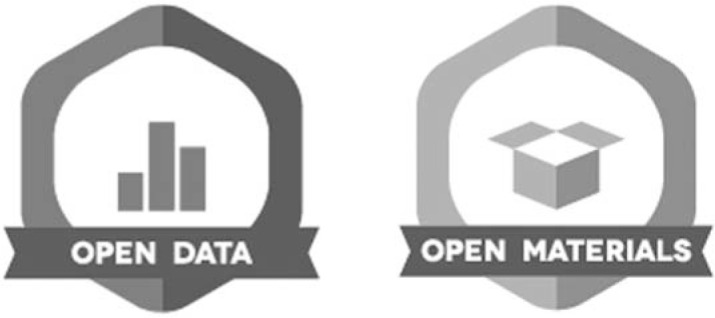


Imagine you want to select some books from an old wooden shelf. While doing so, you notice a spider appearing in a corner at the end of the shelf, which – startled by your activities – directly hides between some of the books. Will attention shift toward that location or divert from that area? And will this affect your immediate actions, for example, when you touch a book in that area? Our study is concerned with this question, namely how phobic stimuli might affect allocation of attention which in turn has a direct consequence for action control processes.

Action control theories assume that when responding to a stimulus, the stimulus features as well as the response are coupled into a short episodic memory trace, referred to as an event file (Frings et al., 2020; Hommel, 2004). If any information repeats, the previous event file is retrieved and affects performance. Fully repeating all information is beneficial because the retrieved event file fully matches. However, if information only partially repeats, interference occurs, resulting in partial repetition costs (Hommel, 1998, 2004; Hommel et al., 2001). Lastly, if no information repeats, there are no benefits nor interference caused by retrieval – because nothing is retrieved. This data pattern can be investigated in prime-probe sequences: In these, participants first respond to a prime target, followed by the response to a probe target. From prime to probe, response repetitions and changes are orthogonally varied with repetitions and changes of response-irrelevant information. The effect resulting from the separate processes of integration into an event file and the retrieval of it (Frings et al., 2020; Laub et al., 2018) is referred to as stimulus-response (S-R) binding.

S-R binding is a strong and stable phenomenon. Binding between responses and (even completely task-irrelevant or response-irrelevant) stimulus information has been observed with features such as location (Hommel, 1998; color discrimination tasks in Schöpper, Hilchey, et al., 2020), color (Hommel, 1998; central target task in Schöpper & Frings, 2022), letters (Frings et al., 2007), sounds (Schöpper, Singh, & Frings, 2020), and more. However, several factors – for example, task type and task setting (e.g., Hilchey et al., 2018; Huffman et al., 2018; Schöpper et al., 2022; Schöpper & Frings, 2022; Schöpper, Hilchey, et al., 2020), target modality (Schöpper & Frings, 2023), or *bottom-up* factors (e.g., perceptual grouping, Laub et al., 2018; figure-ground segmentation, Frings & Rothermund, 2011, 2017; Schmalbrock & Frings, 2022) – can have a modulating role on the occurrence of binding effects by either influencing integration, retrieval, or both (see also Frings et al., 2020, for an overview).

## Impact of Attention on S-R Binding Effects in Action Control

The potential modulating role of attention on integration and retrieval has so far produced mixed results (for a discussion, see Singh et al., 2018). As long as there is some task relevance in feature dimensions, there is only little requirement for attention to features to spur on the integration of such into an event file (Hommel, 2005; Hommel & Colzato, 2004). Yet, attention can have a modulating role on the occurrence of binding effects (see Frings et al., 2020). For example, response-irrelevant stimuli only retrieve the response, if they are attended to (Moeller & Frings, 2014), and binding effects are stronger if the response-irrelevant feature is attended to (Singh et al., 2018). This fits well with the *intentional weighting* mechanism proposed by Memelink and Hommel (2013; see also Hommel et al., 2014): Here, the cognitive system is assumed to assign more *weight* to a certain feature dimension due to task demands (certain instructions or goals, etc.) – like color, location, and so on – and by that, said feature dimension has a stronger impact on processing and performance. Consequently, if attention is directed to certain dimensions, the resulting binding effects increase (Hommel et al., 2001, 2014). To summarize, although the exact impact is debated (see Singh et al., 2018), there have been multiple observations of increased binding effects for attended features, especially if attention is allocated to such features during retrieval (e.g., Moeller & Frings, 2014). In other words, increased attention to a certain feature dimension should increase the strength of observed S-R binding effects.

## Attentional Biases Toward – or Away From – Certain Stimuli

Allocating attention to certain features can be done, for example, by task instructions (e.g., Hommel et al., 2014; Singh et al., 2018). It is also possible to allocate attention toward specific locations, that is, spatial attention, in multiple ways. For example, exogenous attention can be elicited by sudden visual onsets in the periphery and endogenous attention can be elicited by central stimuli that are predictive where the upcoming target will be presented (Posner, 1980; see also Chica et al., 2013, 2014). Attentional effects have been found in response to arrows pointing (e.g., Pratt et al., 2010; Pratt & Hommel, 2003) or eyes looking (e.g., Ristic et al., 2007) somewhere. Moreover, some stimulus categories like highly arousing images (e.g., Vogt et al., 2008) or faces (e.g., Palermo & Rhodes, 2007; Ro et al., 2001) attract attention, for example, when the latter are presented in parallel with nonface objects (e.g., Theeuwes & Van der Stigchel, 2006).

There is a vast amount of research on how specific stimulus categories drive attention in individuals with certain phobias. For example, individuals with high traits of anxiety shift their attention to threatening faces (e.g., Bradley et al., 1998) and threatful scenes (Mogg et al., 2004) more than low trait individuals. Mogg and Bradley (2006) found that individuals with high levels of spider fear have an attentional bias toward spider images presented with short exposure (200 ms), which is no longer observed with longer image exposure (500 ms and 2,000 ms). Spider-phobic individuals are also slower to disengage from such stimuli (Gerdes et al., 2008). However, it is argued that processes in individuals with high fear of spiders follow a pattern of initial vigilance followed by subsequent behavioral avoidance (Pflugshaupt et al., 2005; see also Rinck et al., 2010); this can also result in attentional avoidance of the spider stimulus, as well (e.g., Pflugshaupt et al., 2005; Rinck & Becker, 2006). For example, phobic individuals spend less time on viewing phobia-relevant images, suggesting avoidance behavior (Tolin et al., 1999). This spider avoidance in fearful individuals can occur rather automatically without initial attention toward threat (Huijding et al., 2011). Moreover, Thorpe and Salkovskis (1998) found that spider-phobic individuals do not only attend to spiders but also to regions of safety. In their study, participants had to detect target lights at two positions – a wall and a door. Crucially, they placed a living tarantula at one of these two target locations. Spider-phobic individuals responded faster to an appearing target stimulus, if the location of the real-live spider coincided with the location of the door. Evidence for such parallel scanning for safety in the presence of a real spider has also been supported with eye movement data of spider-phobic individuals (Lange et al., 2004).

To conclude, spider stimuli can initially attract attention (e.g., Mogg & Bradley, 2006); this attentional bias toward the stimulus might switch to or coincide with attentional avoidance away from it (e.g., Pflugshaupt et al., 2005; Rinck & Becker, 2006), especially if there is an alternative indicating safety (Lange et al., 2004; Thorpe & Salkovskis, 1998).

## Current Study

To our knowledge, no previous studies have looked at how the attentional biases generated by spiders might have an impact on binding and retrieval between response and location, especially because retrieval is subject to a potential modulation due to attentional resources. In the current study, participants discriminated the orientation of a shape^1^ which repeated or changed its position. We expected S-R binding between response and location, that is, interference by partial repetitions compared to full repetitions and full changes, evidenced in a crossed data pattern (e.g., Hommel, 2004; discrimination tasks in Schöpper, Hilchey, et al., 2020). Crucially, an image of a cub and a spider appeared between prime target and probe target, one of which was spatially congruent with the subsequent probe target position. If attention to certain features increases S-R binding (e.g., Singh et al., 2018), especially if attention is allocated to such during retrieval (e.g., Moeller & Frings, 2014), we expect stronger S-R binding for trials in which the probe target is spatially congruent with a previously presented spider image due to attentional allocation toward this aversive stimulus category (e.g., Mogg & Bradley, 2006). In contrast, if attentional avoidance of spiders (e.g., Rinck & Becker, 2006) drives the data pattern, that is, attention is shifted away from the aversive content, we expect weaker S-R binding at said position. In any case, an attentional bias or attentional avoidance modulating S-R binding might be especially pronounced for spider-fearful individuals (Rinck & Becker, 2006).

## Methods

### Participants

Binding effects are reliably observed (e.g., Frings et al., 2007; Singh et al., 2016), and those arising from response × location binding can come with very high effect sizes (e.g., *d* = 2.97 and *d* = 2.77 for the color discrimination tasks in Experiment 1 and Experiment 2, respectively, in Schöpper, Hilchey, et al., 2020). One hundred and five students^2^ of the University of Trier participated for partial course credit. One participant was excluded, due to not following task instructions (i.e., pressing only one key for most of the experiment). Three participants were excluded because they were far outliers when compared with the sample (i.e., above 3 × interquartile range above third quartile of number of excluded trials in reaction times and error rates and above 3 × interquartile range above third quartile in overall error rates); these criteria, that is, being a far outlier in several variables, were decided a priori. This led to a final sample size of 101 participants (84 female, 17 male; *M*_Age_ = 21.47, *SD*_Age_ = 2.68, age range: 18–32 years). With assumed α = .05 (one-tailed) and expected effect size of at least *d* = 0.8 for finding a binding effect, this sample size leads to a power of 1 − β = 1.00 (G*Power, version 3.1.9.2; Faul et al., 2007). This sample size is sufficient to find a within modulation of binding due to valence mapping with an expected effect size of *d* = 0.5 (assumed α = .05, two-tailed) with a power of 1 − β = 1.00; moreover, with a power of 1 − β = 0.95, it is sensitive to observe an effect size of at least *d* = 0.36 (assumed α = .05, two-tailed). Prior to experimental start, participants were informed that the experiment involved the presentation of spider images. All participants gave their informed consent to a linked consent form by responding in a text field popping-up at the start of the experiment. The experiment complied with ethical standards for conducting behavioral studies at the University of Trier. One participant reported an uncorrected visual impairment but said participant’s data did not stand out when compared with the sample. Consequently, we included the data. All other participants reported normal or corrected-to-normal vision.^3^

### Apparatus and Materials

The experiment was an online study which was programmed in PsychoPy (Peirce et al., 2019) and then uploaded to Pavlovia (https://pavlovia.org/). Participants were asked to work through the experiment on a computer or laptop, but not on a smartphone or touchpad. Due to the online setup, the exact sizes of stimuli could vary; however, all stimuli, distances, and positions were programmed in pixels so that the relative sizes were identical, irrespective of the device used. At the center of the screen was a white fixation cross (30 × 30 px). Two white frames (length × height: 246 × 186 px) with a line thickness of 3 px appeared 150 px above and 150 px below the fixation cross (center-to-center), that is, frames were 300 px apart (center-to-center). These frames were empty (i.e., black) throughout most of a trial sequence. However, in these frames, an image (240 × 180 px) of a cub (fox cub, polar bear cub, kitten; picture IDs: P081, P095, P096) or a spider (three different species; picture IDs: SP002, SP012, SP100) could appear, all taken from the Geneva affective picture database (GAPED; Dan-Glauser & Scherer, 2011). Targets were gray bars (RGB: 127, 127, 127), which either appeared with a horizontal (80 × 20 px) or vertical (20 × 80 px) orientation. Targets could appear at the center of the upper or lower frame, that is, 150 px above or below the fixation cross (center-to-center).

To measure fear of spiders, we used a short screening developed by Rinck et al. (2002; “Spinnenangst-Screening,” SAS). This short 4-item questionnaire is based on the diagnostic criteria for spider phobia in the fourth edition of the *Diagnostic and Statistical Manual of Mental Disorders* (*DSM-IV*; American Psychiatric Association, 1994). It involves four statements in German language (e.g., “Ich habe Angst vor Spinnen”; I’m afraid of spiders), to which participants have to indicate their agreement on a 7-point Likert scale from 0 (= *trifft gar nicht zu/strongly disagree*) to 6 (= *trifft genau zu/applies exactly*).

### Design

The experiment used a 2 (valence mapping: cub vs. spider) × 2 (response relation: repetition vs. change) × 2 (location relation: repetition vs. change) within-subject design. The binding effect is computed as the interaction of response relation and location relation. Its modulation by valence mapping is derived from the three-way interaction.

### Procedure

**Figure 1:**
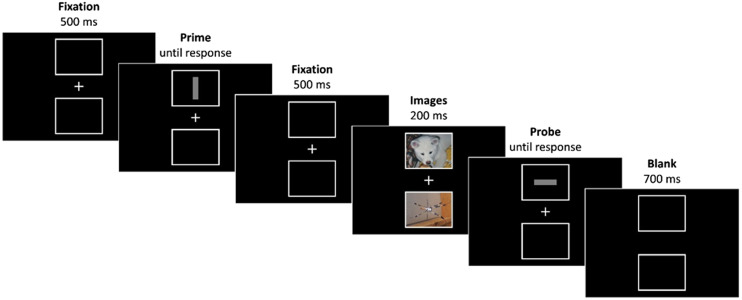
A potential trial sequence (not drawn to scale). This example depicts a trial in which the response changes while the location repeats (RCLR) with cub mapping, that is, the probe target appeared at the position that was previously occupied by an image of a cub. Photographs of animals (by the first author) are only used for illustration; the experiment used images of Dan-Glauser & Scherer (2011).

The experiment took place online; thus, individual settings may have varied. The trial structure was a prime-probe design, that is, participants first gave a response to a prime target followed by a response to a probe target (Figure 1). A trial sequence started with the first fixation display (500 ms), showing the fixation cross at center and an empty white frame above and below it. The prime display was identical except that now a vertical or horizontal bar appeared in one of the two frames until response. Participants were asked to press the F-key for horizontal bars and the J-key for vertical bars. After a response was given, the prime target disappeared, leaving the fixation cross and white frames in isolation again for 500 ms. Now, an image of cub and an image of a spider were depicted in the two frames for 200 ms. There was always one cub paired with one spider, and the possible parings (i.e., fox cub with spider 1, fox cub with spider 2, and so on) were pseudorandomly balanced across the whole experiment. Participants were instructed that the images were task-irrelevant. Directly following the image presentation, the probe target appeared in one of the two frames. Probe responses were given as described for the prime display. After the probe response was given, the probe target and fixation cross both disappeared, leaving the empty frames in isolation (700 ms). If participants responded incorrectly during the prime or probe display, an error message appeared for 1,000 ms directly after the incorrect response.

From prime to probe, the response could repeat with the location of the target also repeating (response repetition, location repetition; RRLR) or changing (response repetition, location change; RRLC), or the response could change with the location of the target repeating (response change, location repetition; RCLR) or also changing (response change, location change; RCLC). Crucially, the positions of the cub image and spider image were orthogonally varied with responses and location. If the position of the probe target was previously occupied by a cub image, this condition was labeled as cub mapping. In contrast, if the position of the probe target was previously occupied by a spider image, this condition was labeled as spider mapping. All combinations of targets, positions, and cub/spider images were pseudorandomly balanced across all conditions and participants. Participants first completed 16 practice trials which were drawn randomly from all combinations. Here participants received feedback for both correct (“Richtig!,” i.e., right) and incorrect (“Falsch!,” i.e., wrong) responses. For experimental trials, participants completed 36 prime-probe sequences for every condition, leading to a total of 288 trials. During the experimental trials, participants only received feedback for incorrect responses. Participants could take self-paced breaks after the 72nd, 144th, and 216th trial.

After the experimental trials, participants completed the 4-item screening for spider fear (Rinck et al., 2002) without time pressure. Responses were given with the number keys.

## Results

Trials in which the prime response was incorrect were excluded from analysis (5.72%) of probe reaction times and error rates. Further, for probe reaction times, we excluded trials if the probe response was incorrect (additional 4.59%), if the probe response was below 200 ms (additional 0.01%), or if it was above 1.5 interquartile range above the third quartile of each individual participant’s distribution (Tukey, 1977; additional 3.62%). Due to these constraints, 13.94% of trials were discarded from the reaction time analysis. Mean reaction times and error rates are depicted in Table 1.

**Table 1:** Mean reaction times in milliseconds and error rates in percent (in brackets) of probe responses as a function of response relation, location relation, and valence mapping

Response relation	Cub mapping		Spider mapping	
	Location repetition	Location change	Location repetition	Location change
Response repetition	520 (1.63)	548 (6.96)	516 (1.87)	543 (6.59)
Response change	571 (8.04)	543 (2.56)	567 (8.41)	539 (3.32)

### Reaction Times

We performed a 2 (valence mapping: cub vs. spider) × 2 (response relation: repetition vs. change) × 2 (location relation: repetition vs. change) repeated measures ANOVA on probe reaction times. The main effect of valence mapping was significant, *F*(1, 100) = 18.42, *p* < .001, $\eta_{p}^{2}$ = .16, with participants responding faster when the previously spatially congruent image was a spider (541 ms) compared to a cub (546 ms). Additionally, there was a main effect of response relation, *F*(1, 100) = 108.60, *p* < .001, $\eta_{p}^{2}$ = .52, in that participants were faster when the response repeated (532 ms) compared to changed (555 ms). The main effect of location relation was not significant, *F*(1, 100) = 0.10, *p* = .753, $\eta_{p}^{2}$ < .01. There was neither an interaction of valence mapping and response relation, *F*(1, 100) = 0.19, *p* = .665, $\eta_{p}^{2}$ < .01, nor of valence mapping and location relation, *F*(1, 100) < .01, *p* = .952, $\eta_{p}^{2}$ = .00. There was an interaction of response relation and location relation, *F*(1, 100) = 388.31, *p* < .001, $\eta_{p}^{2}$ = .80. Response repetitions were faster when location repeated (518 ms) compared to changed (545 ms). In contrast, response changes were faster when location changed (541 ms) compared to repeated (569 ms). These partial repetition costs suggest the occurrence of S-R binding due to location response binding. This interaction was not further modulated by valence mapping, *F*(1, 100) = 0.03, *p* = .860, $\eta_{p}^{2}$ = .00.

**Figure 2:**
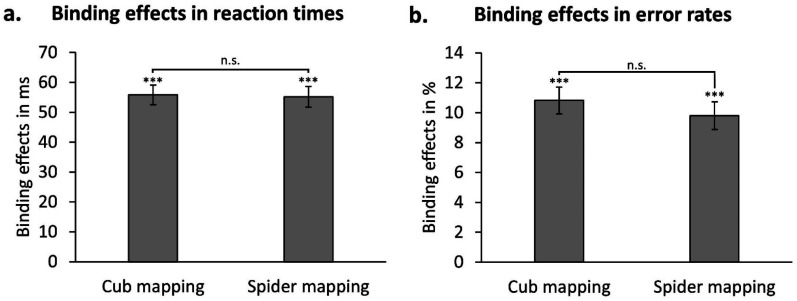
Calculated binding effects separate for cub and spider mappings in (a) reaction times and (b) error rates. Error bars represent standard error of the mean. ***p < .001, n.s. = not significant.

For sake of completeness, we recalculated the interaction of response relation and location relation as (RRLC-RRLR)-(RCLC-RCLR) separate for the cub and spider mapping. This differential value – referred to as the binding effect (c.f., Frings, 2011; Schöpper, Singh, & Frings, 2020; Singh et al., 2016) – adds up the interference of partial repetitions for response repetitions with the interference for partial repetitions for response changes. The binding effect for the cub mapping was 56 ms, *t*(100) = 17.04, *p* < .001, *d* = 1.70, and the binding effect for the spider mapping was 55 ms, *t*(100) = 16.12, *p* < .001, *d* = 1.60. Both binding effects (Figure 2a) did not significantly differ from each other, *t*(100) = 0.18, *p* = .860, *d* = 0.02, and had a BF_01_ = 8.94 (Cauchy prior = 0.707 in JASP; JASP Team, 2023) in favor of the null hypothesis given the data.

### Error Rates

Error rate is the percentage of incorrect probe responses given after correct prime responses. We performed a 2 (valence mapping: cub vs. spider) × 2 (response relation: repetition vs. change) × 2 (location relation: repetition vs. change) repeated measures ANOVA on probe error rates. There was a main effect of response relation, *F*(1, 100) = 12.88, *p* = .001, $\eta_{p}^{2}$ = .11, in that participants made less errors when the response repeated (4.26%) compared to changed (5.58%). Neither the main effects of valence mapping, *F*(1, 100) = 0.90, *p* = .344, $\eta_{p}^{2}$ = .01, and location relation, *F*(1, 100) = 0.21, *p* = .650, $\eta_{p}^{2}$ < .01, nor the interactions of valence mapping and response relation, *F*(1, 100) = 1.63, *p* = .204, $\eta_{p}^{2}$ = .02, and of valence mapping and location relation, *F*(1, 100) = .04, *p* = .836, $\eta_{p}^{2}$ = .00, were significant. Again, the interaction of response relation and location relation was significant, *F*(1, 100) = 170.68, *p* < .001, $\eta_{p}^{2}$ = .63, depicting partial repetitions costs. For response repetitions, participants made less errors when location repeated (1.75%) compared to changed (6.77%). For response changes, participants made less errors when location also changed (2.94%) compared to repeated (8.22%). This interaction was not further modulated by valence mapping, *F*(1, 100) = 1.24, *p* = .268, $\eta_{p}^{2}$ = .01.

The binding effect for the cub mapping was 10.82%, *t*(100) = 12.07, *p* < .001, *d* = 1.20, and the binding effect for the spider mapping was 9.80%, *t*(100) = 10.58, *p* < .001, *d* = 1.05. Both binding effects (Figure 2b) did not significantly differ from each other, *t*(100) = 1.11, *p* = .268, *d* = 0.11, and had a BF_01_ = 4.98 in favor of the null hypothesis given the data.

### Impact of Spider Fear on Binding Effects

We found strong S-R binding effects both in reaction times and error rates. However, an image of a cub or spider spatially congruent with the succeeding probe position did not modulate overall binding effects. We thus looked at the impact of individual spider fear on the strength of binding. From the 4-item questionnaire (Cronbach’s α = .90), we calculated the average value spider fear for each participant, which could range between 0 and 6. A higher value indicates higher self-reported fear of spiders. We then calculated a differential value resembling the three-way-interaction by subtracting the binding effect with spider mapping from the binding effect with cub mapping. Consequently, a positive value indicates a larger binding effect for the cub mapping.

**Figure 3:**
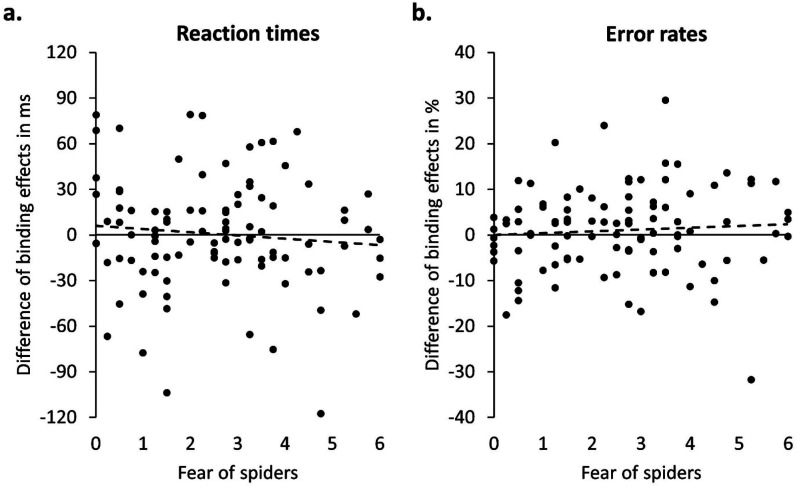
Scatterplots depicting value on fear of spiders questionnaire (*x*-axis) and the differential value of binding effect with cub mapping minus binding effect with spider mapping in percent (*y*-axis) in (a) reaction times and (b) error rates.

To test the hypotheses, we computed Pearson correlation coefficients between spider fear and the differential values for reaction times and error rates. Spider fear did neither correlate with the difference of binding effects in reaction times, *r* = −.09, *p* = .351, BF_01_ = 5.24 (stretched beta prior width = 1.0 in JASP; JASP Team, 2023; Figure 3a), nor with the difference of binding effects in error rates, *r* = .07, *p* = .495, BF_01_ = 6.39 (Figure 3b).

## Discussion

In the current study, participants discriminated the orientation of gray bars, which could repeat or change their position. We found strong S-R binding effects between response and location, both in reaction times and error rates. However, presenting an image of a cub and an image of a spider, one of which was spatially congruent with the probe target position, prior to the probe display did not modulate the strength of binding effects. This effect also did not emerge when taking individual spider fear into account.

When participants respond to stimuli in a sequence, action control theories (e.g., Frings et al., 2020; Hommel, 2004) assume the occurrence of S-R binding. Such binding effects can be pronounced if attention is allocated to certain features (e.g., Hommel et al., 2014; Moeller & Frings, 2014; Singh et al., 2018). In the current study, we aimed to use attentional biases toward (Mogg & Bradley, 2006) or (subsequently) away from (e.g., Pflugshaupt et al., 2005; Rinck & Becker, 2006) spiders, to shift spatial attention to certain locations. In fact, we found participants responding faster to a location at which previously a spider image was presented. This suggests that attention had been shifted toward the aversive content, easing overall responding to a following target if spatially compatible. Yet, this attentional bias toward spider images (e.g., Mogg & Bradley, 2006) did not modulate S-R binding effects. Although higher attentional biases in high fear individuals when confronted with spider stimuli have been observed (e.g., Mogg & Bradley, 2006; Rinck & Becker, 2006), we did not find the effect of interest being modulated by individual fear of spiders.

If spider-phobic individuals are confronted with a spider stimulus, they have been found to attend to the threatful stimulus (e.g., Berdica et al., 2018; Mogg & Bradley, 2006) and subsequently avoid it (e.g., Koster et al., 2006) or even avoid it initially (Huijding et al., 2011). However, other studies have found that highly fearful individuals slower disengage from threatful images (e.g., Georgiou et al., 2005; Gerdes et al., 2008, 2009). Thus, we could have observed a larger S-R binding effect in the spider-congruent position due to an attentional bias toward and slower disengagement from said area or a larger S-R binding effect in the spider-incongruent position due to attentional avoidance of said area. We did not find any modulation. However, the affective images in our study were presented for 200 ms, directly followed – and masked by – the probe target at one of two positions. Thus, probe target onset coincided with the disappearance of affective stimuli, potentially easing attentional disengagement (c.f., Machado-Pinheiro et al., 2013). Moreover, attentional biases toward threat have been found to decrease with time (e.g., Mogg & Bradley, 2006), and attentional avoidance can occur very fast (Koster et al., 2006; Mackintosh & Mathews, 2003); thus, as the probe target appeared until response, that is, on average more than 500 ms after affective image offset, attention may have disengaged and shifted to the other position, reducing the impact of an initial attentional bias toward the spider image. However, future studies might investigate if a longer or even overlapping presentation of affective images with the probe target affects retrieval differently.

In the current study, spider fear was assessed via a short spider fear screening (SAS; Rinck et al., 2002), gearing to the *DSM-IV* criteria for spider phobia, which participants responded to after finishing the experiment. In other words, we did not build our experimental groups of low and high fear based on diagnosed spider phobia. Additionally, prior to experimental start, participants were informed that the study involved spider images; thus, individuals with extremely high fear of spiders or even diagnosed spider phobia might have avoided study participation in the first place. Although the short screening developed by Rinck et al. (2002) showed high internal reliability, we did not find a modulation by spider fear. However, future research might compare group differences between participants with diagnosed spider phobia with an undiagnosed or low-fear sample.

Related to the current study, Colzato et al. (2007) hypothesized that S-R binding is modulated by the dopaminergic system, as the latter is thought to play a role in integration of action features (Schnitzler & Gross, 2005). Based on research that found positive and negative pictures activating and modulating the dopaminergic system (e.g., Dreisbach & Goschke, 2004), the authors hypothesized that this might modulate the strength of binding as well. The authors used a design in which participants gave two responses – one based on a previous cue and the second based on the discrimination of a specific stimulus dimension (see Hommel, 1998, 2004), which was the shape (Experiment 1), location (Experiment 2), or color (Experiment 3) of the stimulus. Crucially, 200 ms prior to the to-be-discriminated stimulus, a positive or negative image appeared at screen center. Congruent with their hypothesis, the authors found increased binding effects for positive image trials, compared to negative image trials; however, this was only found when shape had to be discriminated. In the current study, we did not find larger binding effects for trials with positive mapping; however, note that in contrast to Colzato et al. (2007), who only presented one affective picture in a trial, we presented two pictures of different valence simultaneously (c.f., Berdica et al., 2018; Schöpper, Jerusalem, et al., subm.) – thus, effects by the dopaminergic system might have affected the pattern, but in all trials. Further, attention away from threat to safety (cf. Lange et al., 2004; Thorpe & Salkovskis, 1998) or a general attentional bias toward positive images (for a meta-analysis, see Pool et al., 2016) might have balanced out or at least drastically reduced an attentional bias toward spider images. Therefore, this could be a possible explanation for an absent modulation of attentional biases on retrieval. Future research could modulate attentional biases to positive and negative images differently (for example, by introducing trials with neutral images) to concur this limitation of the current experimental design.

That spider images have diverging (interindividual) consequences concerning attentional biases has been shown multiple times (e.g., Berdica et al., 2018; Rinck & Becker, 2006). Yet, what we here demonstrated is that this kind of attentional weighting of locations due to individual fear of spiders does not have an impact on actions in terms of event file retrieval.

## Footnotes

^1^
We decided to ask participants to discriminate shape/orientation, as this stimulus dimension is prone to be affected by affective information in the context of binding and retrieval (Colzato et al., 2007; see “Discussion” section).

^2^
Testing took place in two separate experiments, with *N* = 35 and *N* = 70, with the second experiment being done to replicate the first experiment (none of the participants participated in both experiments). This procedure was preregistered at https://aspredicted.org/4nt38.pdf. We failed to replicate all findings of Experiment 1. For sake of simplicity and to gain more power for the correlational analysis, we collapsed both experiments to have one large sample.

^3^
For the first experimental session, participants were only asked concerning color blindness, in the second experimental session we also asked for further potential visual impairments.

## Notes

**Publication Ethics:** All participants gave their informed consent to a linked consent form by responding in a text field popping-up at the start of the experiment. The experiment complied with ethical standards for conducting behavioral studies at the University of Trier.

**Open Data:** Data is available at https://doi.org/10.23668/psycharchives.13002 (Schöpper, Küpper, & Frings, 2023a). Code for analysis is available at https://doi.org/10.23668/psycharchives.13003 (Schöpper, Küpper, & Frings, 2023b). Material (stimuli, etc.) is available at https://doi.org/10.23668/psycharchives.13004 (Schöpper, Küpper, & Frings, 2023c).
